# Some Cases of Phthisis Pul.

**Published:** 1892-07

**Authors:** Emil Schlegel


### SOME CASES OF PHTHISIS PULMONUM.

DR. EMIL SCHLEGEL.

[Allg. Hom. Zeit. 19 and 20, 1890.]

During 1876, one of my acquaintances, thirty-six years old, had several times hæmoptæ, and all the characteristic symptoms of pityriasis versicolor, aphonia, and other symptoms. Discharged as incurable from the hospital, he was transferred to the home for consumptives, where he was homœopathically treated, without the least benefit. He read in a newspaper an advertisement looking for an expert in examining and rectifying the books of a large manufacturing establishment. He accepted the post from sheer despair, and started on his mission, and was soon installed in a lone, dilapidated house, half a mile from the village. The office had a stove, but his bed-room was cold, the windows broken, and the wind blew in from all sides. His regular food consisted of milk, eggs, and bread, as nothing else could be had from the village, and water was plenty all around him. He took some cod-liver oil morning and evening. Still, he went to work, though hardly able to stand up, and often, having no companion, the day passed without much speaking. Gradually his strength and appetite increased, and with them a confidence in recovery. Being night and day exposed to fresh air, his chest felt relieved, cough ceased, the voice returned, and when spring came he felt as well as ever. Such a simple mode of living sufficed to create and strengthen the reactive power, and enabled it to banish the demon of phthisis.

A young man of twenty-two years was attacked with hæmoptæ, pleuritic stitches, which left him after three weeks with dyspnœa and copious expectoration; emaciation not yet marked, on the left apex dullness, and moist râles. July 2d, Calcarea-carb.$^{30}$, five powders, to take one every fourth evening. August 3d, by letter: Hardly any cough; feels better; no dyspnœa, nor any expectoration; left eye inflamed; had ophthalmia four years ago; treated by mercurial inunctions. Calcarea brought out again that inflammation, but also a fluent coryza, severe toothache in upper and lower jaw. Belladonna; after two days, Sulphur$^{30}$. December 13th, reports himself well, able to work; the old foot-sweat, which had been suppressed, returned; fauces still injected. Left apex still dull, and breathing not clear. As a constitutional remedy, Thuja, one dose. Since then well. Many such cures Schlegel made during the last twelve years with high potencies (30 and 200), and according to individual indications, he uses most frequently Arsenicum, Belladonna, Bryonia, Calc.-carb., and Hepar, Kali-carb., Kreasot., Phosphor., Sulphur, more rarely Drosera or Stannum.

The rules recommended to consumptives in general are: (1) the patient must be up and dressed the whole day. After dinner (twelve noon) he may keep his siesta. Persons still able or forced to work ought to lie down whenever possible, and on Sundays for several hours; (2) once a week sponging the whole body with tepid water, followed by a thorough rubbing down, and then anointing chest and back with some oily substance, which may be repeated once more during the week. The sponging ought to be done on that day when the patient feels best. Full baths are forbidden. The windows ought to be partially open day and night, particularly the sleeping apartments. Gin-mills and beer-houses ought never to be visited. Wine, beer, and cider are not advisable, or ought to be used only in very small quantities. The patient ought to live on farinaceous food, butter, and bread, leguminosæ, milk, and one or two eggs, daily. Potatoes, roots, fruit are allowable; sugar and fat are highly recommended; salads off and on. The feet ought to be kept warm.

When high-livers descend to such a diet, and stick to it, improvement will follow, when added to it the full use of fresh air and moderate inunctions with fats. Just such a diet, and no other, rouses their vital power, and gives it strength to resist the encroachment of the enemy, and when the necessary medicinal treatment, especially constitutional, is added to it, our prognosis is not any more so ominous.

A few more cases: A tailor, thirty years old, suffered for several months from cough and expectoration, emaciation, chronic hoarseness, diffuse pulmonary symptoms, without much dullness, and night-sweats. After regulating his diet and mode of life (this is always the first requisite, *sine qua non*), he received Phellandrium$^{3}$, glob., thrice daily, five, which he took regularly for six months, when he could be considered able to follow his trade. Numerous ancient physicians praise Phellandrium in pulmonary phthisis, and in many chronic bronchial affections (*Allen's Handbook*, 846), bronchitis, and emphysema, with rapid respiration; cough compels him to sit up day and night, with sleeplessness; ulcers on the legs, with tearing, sticking pains. Valuable for the extremely offensive expectoration in the last stage of phthisis. Farrington gives the same indication. Hughes (706) mentions its ancient use for chronic suppurations in the lungs and elsewhere, and stitching pains in the mammæ. Lilienthal, *Therapeutics*, 863: right lung chiefly affected; cavity, with hissing sound on breathing and horribly offensive expectoration; continuous cough, profuse sweat, diarrhœa, vomiting of food, excessive prostration, and emaciation. Hering (*Guiding Symptoms*, viii), finds it adapted to persons of a feeble, irritable, lymphatic constitution, with weak and defective reaction, and recommends it for bronchial catarrh, where tuberculization is suspected; incessant, persistent cough, excited by a tickling in trachea, and all the other symptoms already mentioned. If only our German brethren would study our American literature, they could unearth many a good hint which now escapes them.

A mason of twenty-nine years was exposed to cold and suffered the penalty. After the usual failure of routine treatment Schlegel found small vesicular râles and weak respiration. Strict phthisical diet. Phosphor$^{30}$, followed by Calcarea$^{30}$ saved him yet, so as to attend to business.

A lady teacher, twenty-four years old, pale and delicate, suffered four weeks ago from pleuro-pneumonia. She is now very emaciated, dyspnœa, dry cough, great lassitude, appetite fair, menses regular. Great dullness of right chest up to the clavicle, back to the spina scapulæ, bronchial breathing, moist râles. Antimonicum-arsenicosum$^{3d}$, which, recommended by several physicians, has been verified for the absorption of right-sided copious exudations, and it acted well in that direction, so that she could be put on Phallandrium and finally on Calcarea-phosphorica, a great antichlorotic remedy in blonde women.

Schlegel gives us some more cases, but he puts the chief momentum on the diet, and now we stand in full accord with Jousset, who strictly forbids all animal food and would rather do without any stimulants. No wonder that phthisis-pulmonalis has been so far unsuccessfully treated as long as we stuff our patient with meat and poultry, though it goes against them, and the patient forces it down because the doctor insists upon it. Schlegel shows in the first case what simple food—milk, eggs, bread and a constant sojourn in the fresh air—will do even in desperate cases, and our chief object ought not to be to treat this or that symptom, but to rouse up the flagging *vis medicatrix naturæ,* and thus enable her to throw off the incubus. Brown-Séquard relates in a late French journal that he relieved and even cured many a case of incipient phthisis by his rejuvenator (he uses now more frequently the testicle of a horse), and thus by giving new strength, the morbid agent is thrown off and the suffering organ relieved. According to all reports Koch's fluid acts in the same manner, while at any rate no poison acts in Brown-Séquard's treatment. We all look forward with great expectation, and still we think that Jousset and Schlegel are on the right road and that the physicians of all schools have to forget the false teachings of their masters and return to the simple diet, fresh air, and sensible exercise to cure their cases. Medication need not be neglected, but it becomes of secondary consideration, and we must not expect much benefit therefrom if we neglect the sanitary and dietary measures so necessary to the patient's welfare. S. L.

